# Child sexual exploitation and abuse: considerations of cultural differences in policy and legislation

**DOI:** 10.3389/fpubh.2025.1721238

**Published:** 2026-03-05

**Authors:** Amy Young, Mazlina Che Mustafa, Norsayyidatina Binti Che Rozubi, Nurul Hasyimah Mat Rani, Yanuar Wismayanti, Delanie Woodlock, Sara Singh, Patrick O’Leary, Michael Salter

**Affiliations:** 1 Childlight East Asia and Pacific Hub, School of Social Sciences, University of New South Wales, Sydney, NSW, Australia; 2 Universiti Pendidikan Sultan Idris, Tanjong Malim, Malaysia; 3 Department of Guidance and Counseling, Faculty of Human Development, Sultan Idris Education University (UPSI), Tanjung Malim, Malaysia; 4 National Research and Innovation Agency (BRIN), Jakarta, Indonesia; 5 Gendered Violence Research Network, Faculty of Arts, Design & Architecture, University of New South Wales, Kensington, NSW, Australia; 6 ARC Centre of Excellence for the Elimination of Violence against Women (CEVAW) and Disrupting Violence Beacon, Griffith University, Brisbane, CA, United States

**Keywords:** child abuse, child sexual exploitation and abuse, cross-cultural responses, technology-facilitated child sexual exploitation and abuse, violence against children

## Abstract

**Introduction:**

Online services have expanded cross-cultural communication worldwide and have facilitated cross-border forms of child sexual exploitation and abuse (CSEA), highlighting cultural differences in conceptualisations of the crime.

**Methods:**

This article uses a case-study methodology to examine how cultural beliefs are integrated into CSEA risk assessments and policy responses in three differing cultural contexts: Malaysia, Indonesia, and Brunei Darussalam. The case-studies contain structured snapshots of policy responses to CSEA in each country and were thematically analysed.

**Results:**

Five key themes were identified across the case-studies: intersectionality, policy dislocation, power and control, child participation and the invisibility of adults.

**Conclusion:**

These areas represent reflection points for policy makers, when conceptualising legislative and policy responses to CSEA, to consider where cultural beliefs may be impacting the development or implementation of responses.

## Introduction

“Childhood means more than just the time between birth and the attainment of adulthood. It refers to the state and condition of a child’s life—to the quality of those years.” (1), p. 357

Violence against children is a worldwide concern. While universalist documents such as the United Nations Convention on the Rights of the Child (UNCRC) provide worldwide guidance on acting within the best interests of children, the interpretation and implementation of this guidance is shaped by cultural influences in nation States (2). The underlying normative and practical assumptions of the UNCRC reflect the circumstances of high-income countries most prominent in its formulation, and do not necessarily map onto the complexities of children’s rights and the governance of their lives in low income or resource constrained contexts. While the UNCRC is the most ratified instrument in the history of international law, Kilkelly (3) argues it is “observed more in the breach than in the implementation” (p. 138). Rights-based frameworks, such as the UNCRC, have been criticised for abstracting children from their social context and promoting human rights as politically neutral and cultureless entities (4, 5). There is therefore a need for a critical analysis of how rights-based doctrines are applied across cultural contexts to prevent harm to children.

The UNCRC contains 54 articles outlining rights to safety, education, play, culture and participation (2). Additional protections are offered by General Comment 25, which outlines the rights of children online to be free from sexual violence, including images of sexual violence or sexual contact with adults, and the right to play, education and rest online. The optional protocol on the sale of children, child prostitution and child pornography (OPSC) is also ratified by 178 States and was developed in response to the Commission of Human Rights insisting on a response to the sexual exploitation of children (6).

This paper unpacks how cultural differences are reflected in child sexual exploitation and abuse (CSEA) policy and legislative responses. Through understanding the role culture plays in risk and response formulation, more effective strategies can be developed and implemented. This paper seeks to understand convergences and divergences between different cultural understandings of CSEA, through comparing national case studies from South-East Asia. Malaysia, Indonesia, and Brunei Darussalam have been chosen as these case studies. This paper specifically addresses the following research questions: (1) what role does culture play in shaping CSEA policy and legislation, and (2) what implications does this have for policy development, especially in regard to responses?

Child sexual exploitation (CSE) policy development and research have been hindered by a lack of clarity in definition and scope (7). This article understands child sexual exploitation as a form of child sexual abuse, with the World Health Organisation (WHO) defining child sexual abuse as the involvement of a child in sexual activity that they do not comprehend, unable to give informed consent to, cannot developmentally consent to, or that violates the laws of society (8, 9). This can be between an adult and child, or between children where one has power, either through age, developmental status, relationship, responsibility or trust over the other. CSE as a subtype of child sexual abuse relates to when a child is coerced, enticed or forced, into engaging in sexual activities believing they will receive something in exchange (10). This element of exchange is of relevance in resource constrained contexts. Ongoing refinement and dialogue regarding consistent definitions of child sexual exploitation and abuse is needed, to ensure all aspects are comprehensively integrated into responses and preventative strategies.

Reviews of intervention mechanisms across Asia highlight that Western-derived models are often poorly suited to the region’s social and cultural realities, calling instead for community-led, culturally sensitive programming that addresses shame, victim-blaming, and cultural taboos surrounding CSEA (11). However, there is limited guidance on how to integrate prompts or questions to understand and unpack the influences of hidden cultural beliefs that may be inadvertently integrated into responses.

## Background

### Operationalising definitions of child sexual exploitation

Despite the increasing global focus on CSEA, the field is marked by ongoing debates and evolving definitions (12, 13). These definitions, which vary significantly across legal, social, and cultural systems, pose a challenge to cross-cultural understanding and comparative research (14). The use of terms such as grooming, child sex work, survival sex, and juvenile prostitution interchangeably or synonymously further complicate the issue (14, 15). International definitions of CSE have evolved over time, reflecting a dynamic and evolving field of research. The 1924 Geneva Declaration on the Rights of the Child asserted that children should be “protected from all forms of sexual exploitation and abuse” (16) but offered no clear distinction between these terms. In 1996, the First World Congress Against the Commercial Sexual Exploitation of Children refined the concept by defining CSE as sexual abuse involving “remuneration in cash or kind” (17).

The United Nations further expanded this definition in 2017 to include “any actual or attempted abuse of position of vulnerability, differential power or trust, for sexual purposes,” with or without financial gain (18). However, these definitions remain subject to interpretation across jurisdictions, influenced by local laws, cultural values, and political contexts.

### Areas of agreement

Despite these broader inconsistencies, several core elements of CSE are consistently identified in international research and practice. These include inducement, grooming, and the sexual gratification of the perpetrator. A systematic review of 74 studies found that all referred to the exchange of sex for resources, whether monetary or non-monetary (14). However, the most common terms used across the studies were found to be prostitution, sex work, or selling sex, terminology which overstates the agency or consent that children can meaningfully exercise in these contexts.

Grooming and the sexual motivation of the offender are widely recognised as central components of CSE, though not always clearly distinguished from other forms of abuse. Grooming was referenced in many studies reviewed by Laird et al. (14), but was often conflated with both CSE and CSA, leading to ambiguity in how it functions within exploitative dynamics (15, 19). Nevertheless, grooming is generally understood as a process, either online or in person, whereby an offender builds trust with a child to facilitate abuse, evade detection, and inhibit disclosure (15). Additionally, while structural factors such as poverty and marginalisation are frequently cited, the offender’s sexual gratification is consistently identified as a primary motivation for CSE. As Laird et al. (14) note, this dynamic remains central to the exploitation process. Despite these areas of apparent convergence, key definitional divergences continue to complicate the field.

Alongside the UNCRC, the Organisation of the Islamic Co-operation (OIC) developed a culturally responsive comparable document to codify the rights of Islamic children, the Covenant on the Rights of the Child in Islam (OICCRCI). Both the OICCRCI (Article 18 part 1) and UNCRC (Article 34) have provisions on the rights on the child to be protected from commercial sexual exploitation, and more generally CSE is an area of agreement between the two documents (20). The UNCRC is endorsed by regional bodies, including the Association of Southeast Asian Nations (ASEAN), in mechanisms through the Plan of Action 2021–2025 (21). This is apt given the focus of this article on three Southeast Asian nations.

### Areas of divergence

Definitions of CSE diverge across jurisdictions due to variations in legal thresholds, cultural norms, and interpretations of consent (22). The first significant point of divergence concerns the age-based criteria for who is considered a “child” or “young person.” While many studies adopt the international standard of under 18 years, others extend the age range to include individuals up to 21 or even 24 years, highlighting the absence of a consistent chronological threshold (14). In addition to numerical cut-offs, definitions often rely on qualitative terms such as child, minor, juvenile, youth, or adolescent, which are not always clear. Cultural, developmental, and legal interpretations shape the term child itself. In some contexts, childhood is determined by developmental markers such as puberty, while in others it is tied to the legal age of majority (23). Cross-cultural and legal differences further complicate matters, as evidenced by the wide variation in age of consent, voting rights, and criminal responsibility across jurisdictions, even within comparable countries such as Australia and the United States (15).

A second central point of divergence concerns whether CSE is explicitly framed as a form of abuse. In Laird’s et al. (14) systematic review of 74 studies, fewer than half incorporated the term abuse into their definitions. Even more concerning, only three studies explicitly included a lack of consent or non-consensual acts as defining features of CSE. This limited attention to consent is striking given that, by definition, sexual activity involving a child cannot be consensual. The omission of explicit abuse reflects deeper conceptual differences and may contribute to inconsistent recognition, measurement, and response to CSE across jurisdictions (24).

Definitions of CSE become particularly contested in minority contexts, where local cultural norms may conflict with dominant legal and social frameworks. For example, under the devadasi system in rural South India, girls as young as six are dedicated to temple priests or patrons for sexual services, often to secure family wellbeing or repay debts (25). In China, prostitution is unlawful but not classified as a crime and engaging in prostitution with an underage girl carries different penalties from rape of a girl under 14 (26). Such cultural and legal variations significantly complicate the recognition, measurement, and response to CSE, highlighting the complexity of the issue. UNICEF’s regional analyses states that universal child-protection models tend to assume individualistic disclosure patterns and standardised reporting pathways, whereas children in collectivist societies face shame, deference to authority, and taboo surrounding sexuality, requiring culturally attuned community-based mechanisms (27). Universal rights-based responses are essential but insufficient without cultural translation, with effective child-protection systems requiring an integration of global standards with locally relevant practices that recognise diverse family structures, cultural stigma, and varied understandings of harm.

### Tools and responses

Despite cross-cultural variations in nature and understandings of CSE, limited tools have been developed or adapted to identify and assess for CSE and its correlates in specific cultural contexts. A systematic review of 15 instruments for early identification of young people at risk of experiencing CSE, for example, found that almost all of the instruments had been developed in English, with the majority of them having originated from the United States (28). Where culturally adapted or translated tools do exist, they tend to look at CSA and child abuse more broadly [e.g., (29, 30)], once again highlighting the conceptual challenges in delineating between CSE and other forms of sexual violence against children. Overall, however, the literature on CSA suggests that there remains a lack of culturally tailored tools. In a review of 52 scales for assessing CSA, and its impacts and correlates, Satapathy et al. (31) found that all of the tools had emerged from North America (and predominantly, the United States). Notably, none of the scales in the review had been developed in Asia, underscoring a persistent gap in culturally specific and responsive tools for CSA assessment.

It is difficult to develop a comprehensive picture of programs for victims of CSE in different countries and cultural contexts—an issue which can be attributed, at least in part, to the limited availability of published research evaluating such programs. Nevertheless, the existing literature draws attention to national and local efforts to respond to the needs and experiences of victims. A 2010 review of program responses to the sexual abuse and exploitation of boys in South Asia, for example, identified child helplines, safe shelters, crisis services, and counselling, as just some of the programs implemented in countries in the region (32). Elsewhere, research by Hounmenou (33) on commercial sexual exploitation of children in West Africa, identified NGOs as playing a considerable role in support services for victims in the region, including the funding of organizations for victim services, as well as the delivery of legal support, healthcare, financial assistance and vocational training.

Taken together, the literature on CSE and cross-cultural contexts highlights discrepancies in how CSE is understood across different cultural settings, as well as gaps in culturally adapted and specific tools for assessing CSE. These discrepancies and gaps have the potential to have implications for identification of and responses to CSE. This paper explores this in greater detail by using a case study approach to examine how culture shapes risks and responses to CSE, and the implications that this has for policy development on the issue. In doing so, the paper aims to emphasise the importance of culturally responsive assessment of and responses to CSE.

## Methodology

This paper applies a child rights framework to understandings of CSEA. Bessel and Gal (4) argue that rights-based frameworks offer a conceptual framework for action through international systems of agreements, through recognition that there are some individual liberties that all humans are entitled to and should be respected across legal and social boundaries (34). The authors acknowledge the limitations of adopting a rights-based framework including that rights can conflict with each other, and are abstract, which lead to contestations when applied (34, 35). The UNCRC and optional protocol were used as tools to assist in analysing the three case-studies selected. These internationally ratified treaties outline best practice towards addressing child sexual abuse across cultures and were thus used as an analytical frame. A rights-based framing brings a focus on non-discrimination. This is apt for the study as it centres on ensuring that responses to CSEA do not discriminate on the basis of culture yet still promote the need for cultural connection.

A case-study approach was applied to allow for comparisons across jurisdictions. Yin (36) argues that a case study approach is applicable:

- if the research is interested in answering a “how” or “why” question,
- when the focus of the inquiry is a contemporary phenomenon in a real-life context,
- when the boundaries of the phenomenon are unclear, and
- when it is necessary to use multiple sources of evidence.

Using Yin’s points as a guide, the case-study approach is suitable to the research questions posed in this paper: (1) what role does culture play in shaping risk and responses to CSEA, and (2) what implications does this have for policy development, risk assessment and responses? These questions were developed using a rights-based framework, highlighting the focus on examining protection, prevention and child wellbeing at the core of the UNCRC. Further, rights-based frameworks focus on accountability. A core tenant of question two is to ensure nation states are being held accountable to develop responses that ensure the best interests of children within their borders.

The approach focuses on the contemporary phenomenon of CSEA in a real-life context, with CSE often having inconsistent definitions across boundaries, making the phenomenon unclear. Multiple sources of evidence are needed to explore the phenomenon of CSE across contexts to understand the role culture plays. This paper acknowledges that CSE overlaps with other types of child sexual abuse, and where appropriate includes these other types of abuse in the case studies. This is also reflective of the lack of clarity on the ways that CSE is included in policy and legislative responses. Case-studies are suited to this study as they allow for the depth of content to be analysed, keeping firm boundaries that can become blurred when using phenomenology, ethnography or narrative approaches. By keeping cases bound it allows for in-depth comparison across context, and a focus on pre-determined themes, rather than utilising more inductive approaches which can bring in unexpected variables, making it difficult to compare across cases. George and Bennett (37) note that case studies are particularly effective for developing and testing theoretical propositions in complex social systems, in this case testing the use of a rights-based framework. This capacity for within-case causal inference offers a methodological advantage over approaches such as thematic analysis or discourse analysis, which offer valuable insights into meanings and narratives but are less equipped to illuminate the contextual conditions under which policy outcomes emerge. Stake (38) argues that case studies produce nuanced, context-specific insights that are especially useful for practitioners and policymakers, offering a level of practical relevance that more abstract qualitative approaches may not achieve.

The case studies included in this paper are Indonesia, Malaysia, Brunei Darussalam. These countries represent nations from the Malay world, with an explicitly Islamic ethos. Their inclusion allows for comparison between three nations with a similar majority ethnic group, religion and positioning in South-East Asia, while also shedding important light on the perspectives and experiences of a region that is underrepresented in academic literature on the topic. There are political, legal and economic structural differences between the three nations. Brunei is a high-income, low population absolute monarchy, while Malaysia is an upper-middle income nation with a federal constitutional monarchy, and Indonesia is a democratic republic with a vast population, categorised as a low-middle income nation. This cultural similarity contrasted with socio-economic diversity allows for conclusions to be drawn on cultural influences on CSEA across case-sites.

The three case studies were constructed from diverse secondary data sources. Table 1 outlines the data sources reviewed for each case-site. Data sources were included from 2010 to 2025.

**Table 1:** Case-site evidence base.

Case-site	Policy sources	Academic sources	NGO and media sources
Indonesia	Indonesia’s violence against children survey SIMFONI-PPA Komnas PA National Police Indonesian Education Monitoring Network	Amalia et al. (50) Bingham et al. (45) O’Leary et al. (51) Putri and Syamsudin (53) Rachmawati et al. (54) Rumble et al. (43) Sari et al. (55) Wismayanti et al. (44) Wismayanti et al. (52)	ECPAT International ECPAT Indonesia UNICEF
Malaysia	Ministry of Women, Family and Community Development Department of social welfare National child protection policy Royal Malaysia Police Malaysian Communications and Multimedia Content Code The Sexual Offences Against Children Act 2017	Attrash-Najjar and Katz (65) Beebeejaun-Muslum (63) Hashim et al. (73) Kavenagh and Maternowska (64) Saharuddin (72) Hussein and Saifuddin (70) Schmidt et al. (61) Trilia and Said (62) Abdul Wahab et al. (69) Weare et al. (66) Zubaidi (60)	ECPAT International ITU UNICEF UN Women WHO
Brunei Darussalam	Brunei Attorney General Brunei’s Children and Young Persons Act Brunei Council on Social Welfare JAPEM Ministry of Social and Family Development	Abdallah and Metussin (78) Choo and Smith (79) Rahamathulla (77) Sulaiman et al. (87) Young (39, 40)	OHCHR United Nations High Commissioner for Human Rights Borneo Bulletin Brunei Times

The cases were constructed using a structured template to guide the authors in bringing together secondary sources. The use of multiple data sources acts as a mechanism of case-study validation, alongside the cross-checking by different authors. The comparative lack of academic focus on Brunei’s child protection responses saw a reliance on Young’s (39, 40) dissertation. As the research topic of the thesis aligns with the focus of this article, and at the time of data collection participants in Young’s (39, 40) dissertation were advised that participation would be used for the dissertation and further academic publications, secondary analysis was judged to be ethical and suitable in this instance. Media sources were also used in Brunei to supplement the lack of available information.

Once the case studies were constructed by authors with expertise in each country, they were reviewed for common themes. Content analysis was used to analyse the data sources, using a deductive coding scheme (41). The deductive coding scheme was developed using the UNCRC as a frame. The codes used have been integrated into headings in the case-studies presented, and the features of the proposed framework outlined in the implications section of this article.

## Case studies

### Case study 1: Indonesia

CSEA remains a highly concerning issue in Indonesia, marked by a high prevalence and severe impacts. As CSEA is a sensitive topic in Indonesian society, it is especially challenging to get accurate prevalence estimates and robust data to inform responses (42). Only a few studies have sought to comprehend the scope and context of violence against children in Indonesia and fewer still have concentrated on CSE (43). According to the findings of a systematic review of literature on CSA in Indonesia (44), most of the research on CSEA in Indonesia uses qualitative approaches based on a case studies specific to small geographic area. As a result, there is no representative data to inform prevalence estimates in Indonesia.

#### Risks for CSEA

Wismayanti et al. (44) examined that public discourse around sex in Indonesia, finding that it is highly constrained, despite advocacy from Indonesian women’s activists on deeply ingrained problems like early marriage and polygamy (45). Silencing of discussions related to sex has made it harder to gather information on child abuse, including CSEA, and necessitated a thorough examination of the 2013 Indonesia Violence Against Children Survey results. Among internet-using children aged 12–17, about 2% reported experiencing “clear examples” of online sexual exploitation or abuse in the previous year (46).

In Indonesia, data from the Ministry of Women’s Empowerment and Child Protection’s (KemenPPPA) Online Information System for Women and Child Protection (SIMFONI-PPA) recorded a total of 28,831 cases of violence against children in Indonesia throughout 2024 (January–December), with sexual violence dominating the abuse experienced by child victims (47). The National Commission for Child Protection (Komnas PA) reported 3,547 child violence complaints in 2023, of which 1,915 cases (over 50%) were sexual violence, highlighting the prevalence of this type of abuse (48). Tragically, a significant portion of these sexual violence cases, approximately 43.31%, occur within the victims’ own homes (49).

In addition, sexual violence in schools in Indonesia also remains a pressing issue. Data from the Indonesian Education Monitoring Network (JPPI) indicates that by September 2024, there were 293 cases of violence in schools, with 42% of these being cases of sexual violence. This not only disrupts the learning process but also leaves long-lasting psychological effects on victims. Amalia et al. (50) also reveals that violence-based education in Indonesian schools is still limited to physical understanding, often neglecting the power dynamics and gender equality that are crucial in preventing sexual and gender-based violence. The absence of this education is risky for adolescents because they often rely on peers for understanding sexuality and sexual violence, which can lead to the further spread of inaccurate and risky information (50). This situation underscores the urgent need for comprehensive prevention, handling, and protection efforts for children in Indonesia.

#### Protective factors

O’Leary et al. (51) assert that Indonesian social work’s commitment to child protection fosters culturally sensitive and socially empowering practices crucial for addressing CSEA. This framework enables social work practitioners to collaborate with families and communities, promoting open discussions about sex, encouraging CSEA reporting, and disseminating information on accessing social services and child protection, ultimately contributing to the prevention of sexual violence. In addition, policy discourses in Indonesia significantly shapes the understanding and representation of CSEA, yet current inconsistencies within these policies impede effective intervention and prevention programs, underscoring the critical need for policy harmony and enhanced community comprehension of the issue (52). Supportive community environments at the village level reduce children’s vulnerability, particularly when combined with public awareness campaigns about child rights and protection (53). Institutional frameworks and community-based child protection programs, such as village-level monitoring and integrated social services, provide formal safeguards and reporting mechanisms that strengthen prevention.

#### Disclosure and response

Limited understandings of CSEA impact disclosure and response rates. The challenges in addressing CSEA in Indonesia are deeply rooted in significant gaps within laws and policies, exacerbated by prevailing cultural taboos and a lack of coordination among stakeholders (52). Thus, addressing CSE effectively necessitates a multi-faceted approach that strongly advocates for collaborative stakeholder engagement, robust legal frameworks, and comprehensive child protection policies. Concurrently, fostering active community involvement is paramount to enhancing awareness, prevention efforts, and the effective handling of cases, ensuring children are protected and supported. Up to 56% of children subjected to online CSEA did not disclose their experiences to anyone due to shame, fear, lack of knowledge about where or how to report, or fears about consequences for family (46).

#### Impacts

Survivors in Indonesia often experience persistent mental health impacts alongside emotional dysregulation, low self-esteem, and disrupted identity formation (54). Socially, victims frequently face stigma, social withdrawal, and difficulty forming trusting relationships, which can impact their education and lead to isolation from their social network (55). Recovery is complicated, with trauma persisting in institutional care or social support settings, highlighting the need for long-term, trauma-informed interventions.

### Case study 2: Malaysia

Malaysia is a multicultural and middle-income country with a population of over 34 million, includes 27.4% children under 18 (56). This demographic profile highlights the urgent need to protect children from risks such as CSEA. The Ministry of Women, Family and Community Development (MWFCD) leads national child protection initiatives, including the Department of Social Welfare (JKM) and the National Child Protection Policy launched in November 2024. Malaysia’s multi-sectoral approach involves MWFCD, the Malaysian Communications and Multimedia Commission (MCMC), law enforcement, and civil society organizations (57). This coordination is critical given that 94% of children aged 12–17 used the internet in 2020, with 4% reporting online sexual exploitation (58). Key legislative measures include the Child Act 2001, Sexual Offences Against Children Act 2017, Communications and Multimedia Act 1998, and the Anti-Trafficking in Persons and Anti-Smuggling of Migrants Act 2007 (ATIPSOM). Despite this robust framework, enforcement remains inconsistent, particularly in rural areas.

#### Risks for CSEA

Digital exposure greatly increases the risk of online child sexual exploitation and abuse (OCSEA). A 2022 survey reported that 4% of Malaysian children using the internet had experienced OCSEA, including blackmail, coercion, or non-consensual image sharing, an estimated 100,000 children annually (59). Girls aged 13 to 15 were most affected (59, 60).

Grooming commonly occurs through platforms like WhatsApp, TikTok, and community forums, where perpetrators exploit children’s trust in digital spaces (59). These interactions are complex to monitor due to encrypted communications and limited parental oversight (61). Low digital literacy, high unsupervised internet use, and cultural taboos around sexuality compound the issue. Restrictive gender norms further discourage children from reporting or seeking help, creating a climate in which exploitation thrives undetected (62).

Social norms significantly shape disclosure. Cultural taboos around sex, especially within conservative or patriarchal contexts, create barriers for victims. Girls often avoid disclosing abuse due to fears of shame or dishonor (63, 64), while boys may fear ridicule or disbelief due to social expectations around masculinity (65, 66). The combined effect leads to underreporting and invisibility (62).

#### Protective factors

Despite risks, several protective mechanisms are in place. The MWFCD offers helplines like Talian Kasih 15,999 and standardized case management for social workers and police (67). NGOs such as P.S. The Children and WAO raise awareness in schools through safe-touch curricula (68). Religious institutions promote child dignity and help shift social narratives around protection.

Law enforcement bodies such as the Malaysian Internet Crime Against Children (MICAC), under the Royal Malaysia Police’s D11 Division, monitor online offenders using the ICACCOPS software to track users accessing child pornography (69).

The Sexual Offences Against Children Act 2017 criminalizes grooming, sexual communication with minors, and child sexual material. Amendments in 2019 allowed law enforcement to intervene earlier in grooming cases (70). The Malaysian Communications and Multimedia Content Code (2022) further enforces content regulation among internet service providers (69).

SUHAKAM, Malaysia’s Human Rights Commission, actively advocates for child protection online. Emphasised that children have the right to live and develop in a safe environment, including in cyberspace. As enshrined in the UNCRC, Article 34, states are obliged to protect children from all forms of sexual exploitation and sexual abuse.

#### Disclosure and response

Barriers to disclosure remain a significant challenge. Taboos, fear of dishonor, and victim-blaming continue to silence survivors (63, 66). Girls may be discouraged by modesty norms, while boys face disbelief or stigma (71). The Malaysian government introduced several legal reforms to deal with CSA cases in 2017, such as (1) the introduction of a specific child sexual abuse legislation, Sexual Offences Against Children Act 2017 (“SOACA 2017”) which was enforced since 10.7.2017, (2) the establishment of specialised court in dealing with CSA cases, and (3) the publication of a guideline known as Special Guidelines for Handling Cases Concerning Sexual Offenses Against Children in Malaysia, which aims to set a uniform approach between Malaysian agencies in dealing with CSA cases.

To improve response, the MWFCD’s initiatives, such as the Talian Kasih helpline and updated child protection policy support earlier and safer disclosures. The Sexual Offences Against Children Act 2017 and its 2019 amendments allow proactive enforcement, reducing dependency on child-initiated reports (70). Digital tools like ICACCOPS enhance proactive surveillance (72). NGOs such as SUHAKAM, P.S. The Children, and WAO provide safe reporting spaces and trauma-informed advocacy. Yet, stigma and inconsistent enforcement still inhibit progress. Expanding disclosure pathways requires community outreach, public awareness, and survivor-cantered services.

#### Impacts of CSEA

CSEA has profound and lasting impacts on children’s mental, social, and emotional development. Survivors may experience depression, anxiety, school dropout, isolation, and low self-esteem (61, 73). These consequences often persist into adulthood, undermining the ability to build relationships, secure employment, and maintain overall well-being (59, 71).

Stigma around sexual abuse often deters recovery, particularly for girls, who may face community and family blame (62). Boys also suffer in silence due to masculine norms and scepticism around male victimhood (71). This results in untreated trauma and lifelong consequences for mental health and social functioning. According to the WHO (74), Malaysia’s readiness to prevent and respond to child maltreatment was below 50%, citing gaps in survivor-centered legal procedures, psychosocial services, and integrated care systems. Although legislative measures have improved, critical gaps in trauma support and enforcement remain.

Malaysia’s legislative and institutional frameworks reflect significant progress, but uneven implementation, cultural taboos, and digital literacy gaps continue to hinder effective protection. Although Malaysia is not short of good legislation, the main problem is reported to lie in its implementation and enforcement (60). Legal reforms and enforcement tools like ICACCOPS and early intervention laws are vital, yet insufficient without broader societal shifts. Inter-agency efforts from the MWFCD, MCMC, police, religious leaders, and NGOs offer a promising model but need sustained investment and community engagement. To effectively combat CSEA, Malaysia must normalize disclosure, expand survivor-cantered services, invest in digital safety education, and uplift marginalized voices in policy reform (68).

### Case study 3: Brunei Darussalam

Negara Brunei Darussalam is a small nation of 459,500 people on the island of Borneo, with 79.4% of the population under 14 years of age (75). Brunei provides a case-study of a nation aiming to address CSEA, across three legal systems (Customary, Civil and Syariah). As a Malay speaking nation with an Islamic identity, there are similarities between Brunei, Malaysia and Indonesia, however there are also unique factors to Brunei, and its pre-Islamic history. As an Islamic Sultanate, Brunei is guided by the philosophy of Malay Islamic Monarchy (MIB), which is taught at every level of education, and integrated into governmental policies.

Brunei has collected little data on child protection (76). The lack of data systems also reflects a lack of definitional categories used by Bruneian policy makers when developing child protection responses. For example, no Bruneian definitions were found for the term “poverty,” “vulnerable children,” “child sexual exploitation,” “sexually explicit content or “sextortion.” Brunei’s Children and Young persons Act defines a child as someone under 14 years of age, and a young person as someone between 14 and 18 (75), however the Islamic Family Law Act defines a child as someone unmarried under the age of 18 qamariah (75). Further contestations between ages can be seen in Brunei’s response to grooming, which can be applied to those under the age of 16 (77).

#### Risks for CSEA

Although Brunei has legal frameworks to protect children, limited implementation and monitoring capacity constrain their effectiveness, leaving children vulnerable to both online and offline forms of sexual exploitation (78). Brunei has the highest internet penetration rate in ASEAN, with 97.5% Bruneians using the internet (77). High internet penetration, coupled with limited digital literacy among youth, increases vulnerability to online grooming, coercion, and exposure to sexual content (78, 79). The introduction of the Child Online Protection National Strategy Framework in 2015, saw the Sultanate define the term child pornography in line with international standards (77). In 2016, the Brunei Council on Social Welfare released a statement examining the effectiveness of child protection legislation in the Sultanate. The government has initiated some good programs geared towards child protection, but many are not fully implemented.

[The lack of] emphasis on children’s issues, resulting in inadequate budgeting, has led to poor implementation…I’m not saying that in Brunei we don’t have programs at all; we do, but in a very limited way and some are very temporary. It only solves the problem for a couple of months…which doesn’t allow total support to the vulnerable children…these children are totally neglected or left to the care of relatives (80).

This critique is echoed by the UN (76), who labelled Brunei’s lack of technical expertise in child protection implementation a challenge. Brunei’s Children and Young Persons Act (81) was modelled on Singaporean legislation, with similarities in the approach to child protection evident. One similarity is in the inclusion of beyond parental control orders in both sets of legislation (82, 83). These offences relate to children who are seen to be acting in a way that is beyond control, or placing themselves in moral danger (82), and can act as a way of criminalising young people who are at risk of experiencing sexual exploitation. While beyond parental control cases are part of Brunei’s civil law code, these cases have increasingly been influenced by cultural beliefs around morality and behaviour, and most commonly involve female adolescents (39, 40). As one child protection practitioners stated in Young’s (39, 40), p. 245 study:

Usually most of them are female that the parents complain they are going out from home for…more than 7 days and then thinking something like alcohol and then having…sexual activities with many partners and everything when they come to us.

Practitioners described these cases as not necessarily illegal but against religious teachings, with young people sentenced to the rehabilitation centre, which is also used to detain young people who have committed other offences, including drug offences (39, 40). By positioning underage sexual activity as an offence, it places the responsibility on the young person, rather than on adults in their lives.

#### Protective factors for CSEA

Young’s (39, 40) work included interviews with child protection specialists in Brunei to understand responses to cases. While no culturally specific tools were noted, the qualitative interviews did highlight ways that practitioners view CSEA in Brunei. For example, CSEA is often linked to sexual promiscuity in adolescence, and a need for sexual education. The quote below illustrates how one practitioner integrates CSEA into sexual education in the Sultanate, with Ezycard referring to phone credit.

“We tell them about what’s happening in Brunei like cases of Ezycard we call this, there is a term called Ezy 5, Ezy 10. Where….the youth, especially girls, they would have this chat group called the Ezy free chat. So Asia free chat, so they would text and they would finds people who would want to give them 25.00 Ezycard or 100 dollars Ezycard, by meeting them up…doing things…and also they would just get the Ezycard…just like that. Not only that, we also have you know this scale of men, they would just hold the cards and put it on their car windows, they would drive around. So the girls or the people who knows about this scheme of this for the Ezycard, they would just take the card and go into the car. We have cases like that… We have cases of Facebook…they don’t tell you their identity so they make the girls fall in love with them and so after that they lure them, … they say we will go out on a date but then after that they rape them (39, 40), p. 230.

Integrating this example into formal sexual education highlights that the burden of protection is being placed on young people and primary prevention in Brunei, rather than focusing on intervening with perpetrators of CSEA.

#### Disclosure and reporting

The Royal Brunei Police Force saw a 30% increase from 2014 to 2018 in CSEA cases in the Sultanate, with 67 cases per year (75). However, there is no system in place to monitor children who have been abused in Brunei (84). In 2014, the Bruneian government released the online child protection framework (85), though no research is available on the risk faced by Bruneian children using the internet. One article, quoting the Brunei Police Force, detailed that 60% of under-16 rape cases were from predators met online (86). It also reinforces the cultural belief in Brunei that children are in need of protection from those “outside” of the family. In interviews with child protection personnel, the belief was evident, as well as with young people when describing times when they have felt unsafe (39, 40). However, practitioners did note that the close-knit extended family network in Brunei can act as a protective factor and conversely can be a risk factor in CSEA cases where there may be multiple adult male family members involved in perpetration (39, 40).

#### Impacts of CSEA

No research has been conducted on the impacts of CSEA in Brunei, however integral research has commenced in the sultanate on the impacts of Adverse Childhood Experiences (ACE) in Brunei. Sulaiman’s et al. (87) work noted that Bruneian adults who had experienced one type of ACE were more likely to engage in substance abuse as a coping strategy in adulthood, and were more likely to suffer further abuse, including sexual abuse, as an adult.

### Case-study summary

Table 2 provides an overview of the similarities and differences between the case-sites.

**Table 2:** Case-study comparisons.

Categories	Indonesia	Malaysia	Brunei
Risk factors	Cultural taboos and silencing of discussions of sex Cultural practices including early marriage School sexual violence Perpetration in the home Power differentials Policy dislocation	Limited parental oversight of encrypted online platform Restrictive gender norms that inhibit disclosure Cultural taboos and silencing of discussions of sex	High internet penetration Weak legislative responses Little data collected to support developing responses Cultural taboos and silencing of discussions of sex
Protective factors	Socio-cultural factors embedded in specialist social work responses Availability of support services	Availability of hotline and support services Legislative strength to enable early intervention in grooming cases	Availability of hotline Introduction of a National Framework on Child Protection 2020 Creation of child protection register in 2022
Disclosure and reporting	Lack of robust reporting data Gendered norms and stigma may inhibit disclosure	Gendered norms Proactive law enforcement to reduce dependency on child witnesses	Limited disclosure and reporting data Gendered norms and stigma may inhibit disclosure
Impacts	Shame, social isolation and stigma	Stigma, which may then result in isolation Responses to trauma are limited due to gaps in survivor-centred legal procedures, psychosocial services, and integrated care systems	Little to no research

## Discussion

The case studies in Indonesia, Malaysia and Brunei highlight similarities and differences in how culture shapes policy responses to CSEA in three jurisdictions that share similar religious and ethnic ties. Reviewing the case-studies, using the UNCRC as a frame enabled gaps in policy and responses to be identified in each jurisdiction, and recommendations to be developed. CSEA responses in all three nations can benefit from robust data systems being implemented and maintained. General Comment No. 13, Article 19 of the UNCRC calls for systematic data collection on all forms of violence against children and that data should be disaggregated (2). For CSEA, this means integrating data systems that monitor trends, understand offending patterns and track effective responses across multiple sectors including health, education, law enforcement and social services.

All three jurisdictions have seen legislation develop to respond to CSEA, however further development is needed in supporting child victims long-term, especially in integrating trauma-informed, shame-sensitive practice, into responses. UNCRC Article 19, protection of children from all forms of violence, requires States to develop recovery and reintegration strategies (2). Article 39 provides further guidance including the need for specialised responses for sexual violence for child victims, trauma-informed counselling and long-term rehabilitation (2). Table 2 highlights that cultural taboos in regards to sexuality and gendered norms inhibit disclosures. This moves beyond implementing systems responses to CSEA by States and instead requires programming responses that address cultural barriers. The impact of cultural values including patriarchal norms, family honour, and stigma around sexuality have previously been found to directly influence children’s willingness to disclose abuse and shape community responses to victims in Indonesia and China (88, 89). This paper also established that these cultural beliefs act as risk factors in Indonesia, Malaysia and Brunei.

Reviewing the case studies highlighted the different interpretations of international children’s rights when implemented into national policies. The lack of culturally specific risk assessment tools noted in all three case studies provides an opportunity for researchers and practitioners to develop culturally responsive frameworks to guide CSEA responses. The three case studies highlighted key areas of inclusion when developing a culturally responsive framework, to assist in identifying where cultural beliefs may be impacting the implementation of effective policies.

### Intersections and marginalisations

All three case studies highlighted that the gender of the child plays a role in risk assessment for CSEA and shaping responses. Intersectional frameworks have long been applied to violence prevention strategies with violence labelled by such frameworks *“*as the conceptual glue that binds” systems of domination together (90), p. 919. Violence, including CSEA, is correlated with socially constructed inequalities, and thus *“*Contextualizing violence reveals how membership in particular race and/or gender groups shapes configurations of violence and how differential group power frames the social understanding of violence” (90), p. 922. Globally, the majority of victims of CSE are female (91), with self-reported data highlighting that girls were more likely globally to self-report CSA than boys (92). Of note for the case studies reviewed, Stoltenborgh et al. (92) also identified that Asia had the lowest rate of self-reported CSA disclosure for girls and boys globally, however, girls (113/1,000) still self-reported in higher numbers than boys (41/1,000). Culture drives gendered beliefs that need to be understood in policy responses, and which see girls at greater risks, and boy’s invisible to responses, including in the case of Brunei to legal remedy where boys have no legal protections from rape (82). Social exclusion is a risk factor for CSE, with young people who face intersecting inequalities at a heightened risk of CSEA (93). The case studies highlighted that children from ethic minority groups will be at risk of social exclusion, and that this needs to be considered in risk assessment and responses (94). This is supported by the UNCRC which contains previsions to urge State actors to act in a non-discriminatory manner on the basis of ethnicity or gender (2).

### Power and control

The case-studies highlighted the way that social power and control can be operationalised by offenders, with the way that social power is shaped by culture needing to be reflectively unpacked by policy makers to ward against unintended consequences. All three case studies are from collectivist societies which impacts disclosure and help-seeking (94), with speaking out about abuse risking the reputation and cohesion in communities. Collectivist cultures generally value social order, harmony and family support, with loyalty and obedience prized (94). These values are not inherently harmful to children, however, need to be recognised as social mechanisms that offenders can use to inhibit disclosures or manipulate situations to promote fears of isolation. This can be a particular risk in grooming behaviours.

### Child voice and disclosure

Social control impacts disclosure of CSE, as does general cultural beliefs regarding child participation in society. This can include how different cultures think about ages of maturity, and how this has been codified in different complementary legal systems. Child participation in a society in general, impacts child disclosure in legal systems and their willingness to disclose in healthcare and educational settings. The value placed on child participation also impacts the willingness of practitioners to invest in strategies to include children in age-appropriate ways across their development. All three case studies highlighted the availability of sexual education for children, adjusted to the religious and moral context, as protective factors, allowing children to develop protection strategies. However, all three case-studies also highlighted challenges in children participating in CSEA responses.

### Complementary policy landscapes

All three nations are signatories to the UNCRC and OICCRCI, however each have codified these rights differently, across not only their child protection policies but intersecting and competing legislations. CSEA responses need to engage across justice, health, communications and education systems, and need to encompass policies at an individual, organisational and community level (95). While there has been some research on cultural considerations for individual and societal responses to CSEA, little focus has been given to cultural responses at a national level, including how nations’ cultural identities may privilege, for example, legal responses to CSE over educational responses. The priorities of government in responding are more frequently shaped by political motivations rather than being evidence-based. Bringing stakeholders from diverse ministries and government together to develop policies that align, rather than compete, to protect young people is essential in developing national strategies that address CSEA.

## Implications

### Framework to guide risk assessment across cultures in CSEA

**Figure 1:**
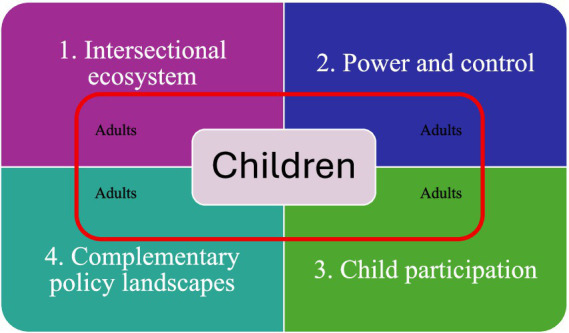
Framework to guide reflection on how cultural beliefs are impacting policy implementation in CSEA cases.

Balancing universality and cultural relativism across jurisdictions is integral to developing effective responses to CSEA. The four themes identified through the review of the case studies have been integrated into a framework, as seen in Figure 1, to guide policy development. This framework allows reflection for policy makers, when conceptualising legislative and policy responses to CSEA, to consider where cultural beliefs may be impacting the development or implementation of responses, especially when young people experiencing abuse have come into contact with the law.

The framework places children at the core of the conversation. This prompts reflection on what cultural beliefs policy makers are holding in regard to “childhood,” including beliefs around children’s capacity, childhood development, children’s responsibility and agency, and what childhood looks like at different ages. Surrounding children in the framework, is an invisible box prompting the policy maker to consider the adults in the case, who are often invisible in assessments. By reflecting on adults when developing policy responses, there is a shift in responsibility from the child and their behaviour to adults. This includes identifying adult perpetrators who have enabled their behaviours, and adults who are protective to partner with the child. Examining the role adults play is a prompt for each of the four reflective boxes, including:

- How are adults impacting the child’s participation in systemic responses? For example, how may they be impacting disclosure?
- How is the power differential between the adult and child impacting the child’s decisions and behaviours? How is coercion and control visible?
- How are the intersectional factors impacting the interactions between adults and children in the case? For example, how may gender or socio-economic status be playing a role in the interactions between adult and child?
- How are other policies or laws privileging the rights of adults over the rights of the child?

The four categories are reflection points, encouraging policy makers to deepen their engagement with important cultural beliefs that may be impacting the effectiveness of responses. They have been intentionally positioned to overlap and complement when read in a clockwise direction, commencing with intersectionality and marginalisation. This first box prompts reflection on how gender, socio-economic marginalisation, ethnicity and disability (as examples) need to be considered in the policy development process. Example prompts include:

- How are the practitioners, adult perpetrators and child’s beliefs in regards to gender shaping the outcomes in CSEA cases? How would we be responding if the gender of the child were different?

Intersectionality aligns with power dynamics in society, and therefore understands that particular genders, ethnic groups and economic statuses hold more power and privilege than others. Understanding power dynamics (as reflected in the second box), and their role in coercion and social control, are core to recognising CSE. This is particularly difficult when offenders engage in image management, and/or engage system responses as part of the abuse. Prompts to reflect on the role power, at individual, community and structural levels are playing can include:

- How may an offender be using social and cultural beliefs to control not only the child but also the child’s family, and community?

Power differentials between adult and child can be powerful tools of silencing. As can be seen in the case studies, collectivist societies may value the wellbeing of the community over the wellbeing of an individual, which can be used as a way of silencing disclosures of abuse. An understanding of how marginalisation, power dynamics and the positioning of children in society cumulatively impacts on children’s voices in the process of disclosure is crucial to effective policy development. Child participation in society (see the third box) is integral in the UNCRC and OICRRCI, however, active participation in society is often not facilitated due to beliefs about the role of children. Child participation reflection prompts can include:

- How has the child actively been engaged in the process? Is this developmentally appropriate? How could the process have been different if child participation had been centred?

The final reflection prompt focuses on how other systems, with competing focuses, impact outcomes (see the fourth box). Policies and legislation focusing on health, education, justice, communications and online safety all impact CSE responses, yet often do not have specialist knowledge on child violence or abuse. Prompts to reflect on how systems can complement and support CSE responses include:

- How is the information collected from other systems supporting or conflicting with desired outcomes? What belief systems underpin these systems? Are their aims and missions complementary to CSE responses and the best interests of the child?

The outlined framework can also inform the practice of INGOs UNICEF and ECPAT as they focus on ensuring nation states meet their international child rights commitments. The prompts applied with curiosity can assist INGO programs to better detect culturally masked sexual exploitation, that has been systemically integrated into local responses. It can also assist in further integrating a focus on adult offenders, largely absent from INGO child protection responses. CSEA responses must move beyond focusing solely on children’s vulnerabilities and instead make adult offenders and facilitators visible within prevention, policy, and accountability frameworks (59, 96). Making adult offenders visible shifts the CSEA narrative from child risk to adult accountability. This aligns with a child-rights framework, as it facilitates child protection and participation.

### Limitations

This paper constructed three case studies from expert understandings of CSEA in Indonesia, Brunei Darussalam and Malaysia. It is a limitation of the study that only three case studies were constructed and that they are all from nations in South-East Asia. Further research can take a greater international focus, enabling greater comparison between cultural and national contexts, and developing a deeper understanding of the impact of culture on CSEA risks and protections. A further limitation of the study is the lack of rigorous child protection studies undertaken in Brunei Darussalam. This contributed to the Brunei case-study being less developed than the Malaysian and Indonesian case-studies, impacting generalisations made across case-studies. This paper calls for greater academic and NGO attention on child protection in the Bruneian context. Nevertheless, this article offers an integral perspective from underrepresented nations in academic literature. There was limited publicly available information on CSE in all three nations, which saw the focus widen to CSEA. This suggests a greater need for rigorous research specifically into CSE in the Global South.

## Conclusion

How countries and individuals respond to CSEA is tied to cultural beliefs around childhood, sexual morality, gender, power, and participation in society. This paper identified a need for the development of risk assessment tools that are culturally responsive, to ensure that responses are appropriate in different countries and cultural contexts, while still aligning with nations’ international child rights commitments. A framework was proposed to guide standardised risk assessment tools. Further research is needed in understanding sexual exploitation as a sub-type of CSA cross-culturally and to promote intercultural dialogue on CSE. This article proposes a culturally responsive framework for policy makers, when conceptualising legislative and policy responses to CSEA, to consider where cultural beliefs may be impacting the development or implementation of responses.

## Data Availability

The original contributions presented in the study are included in the article, further inquiries can be directed to the corresponding author/s.
